# Prevalence of work related musculoskeletal disorders in Italian workers: is there an underestimation of the related occupational risk factors?

**DOI:** 10.1186/s12891-020-03742-z

**Published:** 2020-11-12

**Authors:** Fabrizio Russo, Cristina Di Tecco, Luca Fontana, Giovanna Adamo, Adriano Papale, Vincenzo Denaro, Sergio Iavicoli

**Affiliations:** 1 Department of Orthopaedic and Trauma Surgery, Campus Bio-Medico University of Rome, Via Alvaro del Portillo 200, 00128 Rome, Italy; 2 Department of Occupational and Environmental Medicine, Epidemiology and Hygiene, Italian Workers’ Compensation Authority (INAIL), Monte Porzio Catone, 00078 Rome, Italy; 3 Department of Public Health, Section of Occupational Medicine, University of Naples Federico II, Via S. Pansini 5, 80131 Naples, Italy; 4 Department of Public Health and Infectious Diseases, Sapienza University of Rome, Piazzale Aldo Moro 5, 00185 Rome, Italy

**Keywords:** Musculoskeletal disorders, Low back pain, Upper limbs, Lower limbs, Biomechanical risk, Ergonomic risk, Video display unit risk, Health surveillance program, Risk assessment

## Abstract

**Background:**

Work-related musculoskeletal disorders (WMSDs) represent an important socio-economic burden. The current risk assessment and management involved in the ethiopathogenesis of WMSDs is based on observational tools and checklists, which have some limitations in terms of accuracy and reliability. The aim of this study was to assess WMSD prevalence and identify possible correlations with several socio-demographic and work-related variables in a large cohort representative of Italian workers in order to improve our understanding of the WMSD phenomenon.

**Methods:**

This study includes data from INSuLa, a cross-sectional nationally representative survey of health and safety at work, developed by the Italian Workers’ Compensation Authority. A total of 8000 Italian workers were included. Multivariate logistic regression analyses were performed to evaluate the association of independent variables, such as workers’ perceptions of exposure to biomechanical/ergonomic and video display unit (VDU) risks (Risk Perceived) and the actual risk exposure (Risk Detected) on Back, Lower and Upper limb pain. Socio-demographic, occupational and other health-related variables were included to investigate possible association with musculoskeletal disorders.

**Results:**

Workers perceiving a significant exposure to biomechanical/ergonomic and VDU risks but not included in a health surveillance program for them (Risk Perceived/No Risk Detected) have had significantly higher odds of reporting musculoskeletal disorders. Regarding the biomechanical/ergonomic risk these workers are in the 19–24 age range (39.9%), transportation, warehousing/information and communication sectors (38.9%) and are employed in companies with more than 250 workers (35.8%). Regarding VDU risk, workers are in the 45–54 age range (24.5%), professional, financial and business services (38.0%) and come from companies with more than 250 employees (25.6%).

**Conclusions:**

Within the occupational safety and health management systems an appropriate assessment of occupational risk factors correlated to musculoskeletal disorders (mainly biomechanical/ergonomic and VDU) and the correct definition of their exposure levels is essential to adequately prevent the onset of WMSDs. In this regard, our findings provide useful information to design novel approaches, aimed at improving our understanding of emerging risks, identifying gaps in current risk assessment strategies and enhancing workplace interventions are mandatory to improve the occupational risk assessment and management process and therefore implement the subsequent health surveillance systems.

## Introduction

Musculoskeletal disorders (MSDs) represent a significant worldwide health problem with important socio-economic consequences. Indeed, they affect about a third of the worldwide population representing one of the most important causes of chronic disability, sick leave absence, reduced work productivity and quality of life [1]. Data provided by the 2017 Global Burden Disease study showed that MSDs were the highest contributor to global disability (16% of all years lived with disability - YLDs), and low back pain remained the single leading cause of disability since 1990 [2]. Interestingly, the available literature data demonstrated that the prevalence of these disorders in specific working populations and/or occupational sectors is significantly higher than in general population [3] showing a causal relationship between different types of occupational risk factors (e.g. awkward positions, repetitive movements, low temperatures, manual handling of heavy loads, prolonged computer work, mechanical vibrations, work-related stress) and the development of MSDs that, in this context, are defined as Work related Musculoskeletal Disorders (WMSDs) [4–7].

Although the trend over the last few years [8, 9] has shown a slight decrease in workers complaining WMSDs, it is worth noting that recent reports by the European Agency for Safety and Health at Work (EU-OSHA) showed that more than half of European workers still face this health problem. In Italy the percentage of the workforce declaring one or more WMSDs has significantly decreased from 65% in 2010 to 50% in 2015 [8, 9]. Nevertheless, these disorders are the most common occupational diseases, representing the 66.7% of all Italian occupational diseases recognized in 2018 [10] with back pain being the most commonly identified health problem, followed by muscular pain in the upper and lower limbs (51.6%, 46,7 and 29.3%, respectively) [11]. Soft tissue diseases and dorsopathies are the two most preponderant WMSD types in Italy [12].

The risk assessment and management process of occupational risk factors involved in the ethiopathogenesis of WMSDs such as awkward positions, repetitive movements, manual handling of heavy loads and prolonged computer work is mainly based on the use of observational strategies, tools and checklists [13, 14]. For example, in the risk evaluation of manual handling of heavy loads the Directive 90/269/EEC has indicated as reference methodology the technical standards of the International Organization for Standardization (ISO) 11,228 series, which in turn adopt several well-identified analysis methods [15]. However, in this regard, it should be noted that, an accurate analysis of ISO ergonomics standards in terms of biomechanical load assessment has raised several critical issues [16]. Moreover, the assessment of awkward positions is often carried out when this type of risk is associated with other occupational risk factors, especially when a condition of prolonged computer work occurs. In this regard, the Directive 90/270/EEC on risk related to Video Display Unit (VDU) use at workplace highlighted that working with a VDU for more than 20 h per week represents a significant risk condition that need the activation of health surveillance system [17]. The length of the working period at VDU is generally assessed through self-administered questionnaires or interviews based on a checklist. Unfortunately, this subjective assessment of the time required to perform VDU tasks has critical issues (e.g., evident discretion, poor accuracy in estimating usage time of mouse or keyboard, poor objectivity) which could lead to a possible overestimation or underestimation of the risk [18].

Therefore, the design and consequent implementation of suitable prevention measures must necessarily be based on accurate and reliable quantitative information of high quality which highlight the real dimensions of this topic, without underestimating WMSD prevalence.

In this regard, the aim of the study was to evaluate the prevalence of MSDs and to investigate potential associations with different socio-demographic and work-related variables in Italian workers [11]. This was investigated both in those workers actually subjected to health surveillance programs (as exposed to manual handling of loads, repetitive movements and/or fixed and awkward postures) and in those with a high level of self-perceived biomechanical/ergonomic risk but not subjected to health surveillance medical examinations. These data could be useful in identifying any research areas in which further studies should be carried out to improve our knowledge and understanding of the WMSD phenomenon.

## Methods

### Study population and survey procedure

This study was based on data from a cross-sectional nationally representative survey of the Italian workers population, named INSuLa (INAIL, 2014), developed in 2013 by the Italian Workers’ Compensation Authority to investigate the health and safety at work. INSuLa counts 8000 Italian workers aged from 16 to 64 years. Data were collected during the period from July to December 2013 through structured interviews, using the Computer-assisted telephone interviewing (CATI) method. Sampling strategy was developed to provide a representative sample of the entire national workforce, excluding self-employed, military and civil protection personnel that have different and special applications of occupational health and safety (OSH) legal framework. Representative data on around 17,000 workers from the 2012 National Labour Force Survey from the Italian Institute for Statistics (ISTAT) were used to obtain the selection criteria useful for delineating the universe of interest. A quota sampling strategy was applied by calculating the strata and the quota for the following characteristics: regions, gender and age, type of contract, level of employment, and sector of activity. Eligible persons were reached through a random procedure applied via telephone (random digit dial). A random sample was drawn by verifying the eligibility conditions of the respondents, and respondents were classified according to the stratification characteristics. Sampling was continued up to the fixed proportion of each strata was reached, and a weighting was applied to reach the proportion of the source population exactly.

### Measures

The standardized questionnaire used to conduct interviews was developed after a literature review and a benchmarking analysis of the most prominent European surveys and tools in the field. Questions investigated the main aspects related to health and safety at work in terms of working conditions, risk exposure and perceptions, health status and outcomes, management and prevention actions, role of occupational health and safety professionals and perceptions of the legal requirements.

A description of the variables included into this study and how these were treated by researchers follows into this paragraph.

### Musculoskeletal diseases

Variables measuring the occurrence of musculoskeletal diseases (MSDs) came from a questionnaire sections focussing on health conditions. Participants reported to have had or not some diseases and/or chronic conditions linked to three main areas of the musculoskeletal apparatus, namely: 1. Back, 2. Lower limbs (hips, legs, knees, feet, etc.), and 3. Shoulders, neck and/or upper limbs. Questions refer to the last 12 months frame time and provide a dichotomous answer (yes/no), including the possibility of reporting “don’t know”. Back, Lower limbs and Shoulders, neck and/or upper limbs are included as outcomes related to MSDs into this study.

### Risk perceived vs risk detected

According to the scope of this work, we included two measures aiming at collecting information about risks for health and safety at work that are generally considered as potential predictors of MSDs, respectively Biomechanical and Ergonomic risk (e.g. manual handling of loads and awkward work postures) and VDU risk.

In particular, it was decided to focus on two different aspects: 1) the workers’ perceptions of risk exposure (Risk Perceived) and 2) the actual risk exposure, which is proven by the inclusion of a worker in a health surveillance programme after a systematic assessment of exposed or potentially exposed to occupational hazards (Risk Detected).

As regard to the Risk Perceived, we selected questions aiming to investigate the workers’ perceptions of each kind of risk (Biomechanical and Ergonomic/ VDU risk) asking: “How much do you feel exposed to this risk?” with a response scale from 0 (not exposed at all) to 10 (completely exposed). For each of the two risks, we calculated percentiles to identify the percentage of scores that fall below the 50th percentile as cut off in the sample distribution. Accordingly, the two variables were recorded as dummies by assigning 0 to the scores below the 50th percentile as “No= not risk perceived”, and 1 to the scores beyond the 50th percentile as “Yes = risk perceived”. These new variables were named “Biomechanical and Ergonomic Risk Perceived” and “VDU Risk Perceived”.

For identifying the Risk Detected, we focussed on a question asking for each risk (Biomechanical and Ergonomic risk/ VDU risk): “Are you included in a health surveillance programme due to a verified exposure to risk at work?” with a dichotomous answer (Yes/No).

Crossing the variables presented above, we created a new variable named “Risk Perceived vs Risk Detected” respectively for the Biomechanical and Ergonomic risk and VDU risk. On the basis of the crossing in answers of Risk Perceived and Risk Detected, this new variable is constituted by 4 groups of answers: 1) No Risk Perceived/No Risk Detected; 2) Risk Perceived /Risk Detected; 3) No Risk Perceived/Risk Detected; 4) Risk Perceived /No Risk Detected.

### Occupational and other health related variables

Some occupational characteristics reported by the participants were included into this study to investigate their possible concurrent effects on MSDs. The occupational sector based on the nine categories from the National industrial classification of all economic activities (ATECO), the occupational position (top and middle manager, white collar, blue collar, apprentice or other), the type of contract (permanent, fixed-term or temporary), occupational tenure (years of experience) shift work (yes/no), night work (no, 1 to 2 times a week, more than 2 times a week), working hours (usual number of hours worked per week in the last 6 months); the firm size (4 categories 1 to 9 employees, 10–49 employees, 50–249 employees and more than 250 employees).

As regard to health related variables, stress, anxiety, depression and insomnia in the last 12 months were included (yes/no). The body mass index (BMI) was included by using the person’s height and weight (height/weight^2^) and it was interpreted with the cut-offs identified by the World Health Organization (overweight = BMI ≥ 25 kg/m2 and obesity = BMI ≥ 30 kg/m2). Finally, information related to the actions done by the organizations to raise awareness on health and safety management were included such as two questions investigating the provision of information on health and safety at work (yes/no) and the existence of training programs on this topic (yes/no).

### Control variables

Information collected by the participants included gender, age in years and education level (lower secondary, upper secondary, graduate and post graduate) and BMI (underweight, normal, overweight, obese).

### Statistical analysis

Three separate multivariable logistic regression analyses were carried out to evaluate the association of the independent variables included in the study and each of the three MSDs treated as binary outcomes (namely Back, Lower limbs and Shoulders, neck and/or upper limbs). A manual backward stepwise approach was used to select variables to be included in the final three models [19, 20]. Specifically, as a first step a univariate model was run to select variables to be preliminary excluded for each of the outcomes, using a *P*-value of 0.200. As a second step, all variables emerging with a *P*-value of less than 0.200 in the univariate analysis were included in a multivariable model, where the *P-*value was set to 0.100. All the variables with a *P-*value more than 0.100 were thus eliminated. As last step, a final multivariable model, one for each outcome, was run with a *p*-value set to 0.050. Likelihood ratio test was used to calculate *P*-values and to select variables to be included in the nested models. Odds-ratios (OR) were used as the measure of association. Age, sex, education level and BMI were included as control variables. Missing data were handled with a list-wise deletion approach and only participants with complete information for all exposures and outcomes were included in the final analyses. Finally, a Chi-square test was performed and adjusted standardized residuals were used to measure the strength of the difference between observed and expected values in order to investigate characteristics of the 4 groups emerged by crossing Risk Perceived and Risk Detected (for both Biomechanical and Ergonomic and VDU risks) in relation to age, occupational sector and firm size. The STATA V.15.1 statistical package was used for all analyses.

## Results

### Sample characteristics overview

Descriptive statistics are reported in Table 1. The mean age of the 8000 respondents was 43.0 + − 9.8 years (ranging from 19 to 64 years), comprising 4313 male subjects (53.9%) and 3685 female subjects (46.1%). Most of the participants (52.3%) attended high schools, while only 25.0% had a university education. The prevalence of overweight and obesity was respectively 37.6 and 8.4%. Most representative occupational sector was the Manufacturing/industry sector (23.3%), followed by Commerce (17.6%) and Education (15.1%). A higher proportion of participants were either white (41.7%) or blue collars (45.7%); 85.0% had a permanent work contract, 70.1% had more than 15 years of experience and 39.2% worked in large companies. The majority of respondents (62.3%) worked between 35 and 40 h per week and had a regular working schedule (67.6%), while only a minority are night shifters (8.5%). A large proportion of participants (45.4%) reported to feel stressed at work. On the contrary, only a minority reported to suffer from anxiety (17.5%), depression (7.7%) and insomnia (25.4%). Most of participants received information (88.0%) or training (76.6%) to improve competence on health and safety at work. Almost 12% of the sample reported to feel exposed to ergonomic risk and to receive periodically a medical check for this risk. Remarkably, a larger proportion (30.0%) reported to feel exposed to ergonomic risk without receiving an objective risk assessment. Similar proportions were found among those reporting to feel exposed to VDU risk and those reporting to receive periodically a medical check for this risk (23.5 and 23.7%, respectively).

**Table 1:** Characteristics of study population

Variable	N	%
***Sociodemographic characteristics***		
Sex		
Males	4313	53.9%
Females	3685	46.1%
Age		
19–24	376	4.7%
25–34	1543	19.3%
35–44	2566	32.1%
45–54	2413	30.2%
55–64	1100	13.8%
Educational level		
Lower secondary	1741	21.8%
Upper secondary	4184	52.3%
Graduate and post graduate	2003	25.0%
Do not answer	70	0.9%
BMI		
Underweight	169	2.1%
Normal	4153	51.9%
Overweight	3007	37.6%
Obese	669	8.4%
***Occupational characteristics***		
Occupational sector		
Agriculture, fishing, and hunting	171	2.1%
Manufacturing/Primary industry/Mining/Utilities	1867	23.3%
Construction	435	5.4%
Wholesale and retail trade/Automotive and motorcycle repair/Accommodation and food services	1407	17.6%
Transportation and warehousing/Information and communication	649	8.1%
Professional, financial and business services	950	11.9%
Healthcare and social assistance	708	8.9%
Education services/Public administration, social security	1206	15.1%
Other public and personal services	605	7.6%
Occupational position		
Top and middle manager	740	9.3%
White collar	3334	41.7%
Blue collar	3653	45.7%
Apprentice or other type of employment	271	3.4%
Type of contract		
Permanent job contract	6796	85.0%
Temporary job contract	815	10.2%
Others	387	4.8%
Firm size		
1 to 9	1251	15.6%
10 to 49	1566	19.6%
50–249	1713	21.4%
≥ 250	3136	39.2%
Do not know	332	4.2%
Working hours		
1–34 h/week	1864	23.3%
35–40 h/week	5004	62.6%
41–48 h/week	700	8.8%
49–54 h/week	265	3.3%
> =55 h hours/week	165	2.1%
Shift work		
Yes	2588	32.4%
No	5410	67.6%
Night shifts		
Never	7317	91.5%
1 to 2 times/week	426	5.3%
> 2 times/week	255	3.2%
Work experience		
< 1 year	49	0.6%
1–5 years	358	4.5%
6–10 years	872	10.9%
11–15 years	1114	13.9%
> 15 years	5605	70.1%
***Risk perception and health surveillance at work***		
Biomechanical and Ergonomic risk		
No Risk Perceived/No Risk Detected	3928	49.1%
Risk Perceived /Risk Detected	935	11.7%
No Risk Perceived/Risk Detected	498	6.2%
Risk Perceived /No Risk Detected	2639	33.0%
Video Display Terminal risk		
No Risk Perceived/No Risk Detected	3586	44.8%
Risk Perceived /Risk Detected	1879	23.5%
No Risk Perceived/Risk Detected	636	8.0%
Risk Perceived /No Risk Detected	1899	23.7%
***Other health-related aspects***		
Work-related stress risk		
low	2035	25.4%
moderate	2331	29.1%
high	1650	20.6%
very high	1982	24.8%
Anxiety		
Yes	1396	17.5%
No	6595	82.4%
Do not know	7	0.1%
Depression		
Yes	615	7.7%
No	7383	92.3%
Insomnia		
Yes	2033	25.4%
No	5961	74.5%
Do not know	4	0.1%
Information on health and safety at work		
Yes	7038	88.0%
No	960	12.0%
Training on health and safety at work		
Yes	6131	76.6%
No	1867	23.3%
**Total**	**8000**	

The 12-month period prevalence of MSDs was 51.0% for Back pain, 46.1% for Shoulders, neck and/or upper limbs and 28.6% for Lower limbs.

Findings from the final multivariable logistic regression models for Back, Upper limbs and Shoulders, neck and/or upper limbs are presented in Tables 2, 3 and 4, respectively.

**Table 2:** Multivariate logistic regression analysis assessing factors associated with the occurrence of Back pain in a representative sample of 8000 Italian workers

Variable	OR	95% CI
Sex		
Male	ref	
Female	1.45	1.31–1.61
Age		
19–24	0.68	0.54–0.87
25–34	0.94	0.82–1.08
35–44	ref	
45–54	1.01	0.89–1.14
55–64	1.32	1.13–1.55
Educational level		
Lower secondary	ref	
Upper secondary	0.78	0.68–0.88
Graduate and post graduate	0.71	0.61–0.83
BMI		
Underweight	1.06	0.76–1.48
Normal	ref	
Overweight	1.25	1.12–1.39
Obese	1.29	1.08–1.55
Biomechanical and Ergonomic risk		
No Risk Perceived/No Risk Detected	ref	
Risk Perceived /Risk Detected	1.91	1.62–2.24
No Risk Perceived/Risk Detected	1.04	0.85–1.27
Risk Perceived /No Risk Detected	1.63	1.46–1.82
Video Display Terminal risk		
No Risk Perceived/No Risk Detected	ref	
Risk Perceived /Risk Detected	0.97	0.86–1.11
No Risk Perceived/Risk Detected	0.67	0.55–0.81
Risk Perceived /No Risk Detected	0.95	0.84–1.08
Work-related stress		
Low	ref	
Moderate	1.44	1.26–1.64
High	1.50	1.30–1.74
Very high	2.03	1.75–2.36
Anxiety		
No	ref	
Yes	1.50	1.31–1.73
Insomnia		
No	ref	
Yes	1.78	1.59–2.00
Training on health and safety at work		
Yes	0.84	0.75–0.94
No	ref

**Table 3:** Multivariate logistic regression analysis assessing factors associated with the occurrence of Lower limb pain in a representative sample of 8000 Italian workers

Variable	OR	95% CI
Sex		
Male	ref	
Female	1.23	1.08–1.41
Age		
19–24	0.91	0.69–1.20
25–34	0.99	0.84–1.16
35–44	ref	
45–54	1.38	1.21–1.58
55–64	1.95	1.64–2.32
Educational level		
Lower secondary	ref	
Upper secondary	0.78	0.67–0.90
Graduate and post graduate	0.68	0.56–0.82
BMI		
Underweight	1.07	0.74–1.55
Normal	ref	
Overweight	1.38	1.22–1.55
Obese	2.37	1.96–2.86
Occupational sector		
Agriculture, fishing, and hunting	0.98	0.67–1.44
Manufacturing/Primary industry/Mining/Utilities	ref	
Construction	1.05	0.81–1.36
Wholesale and retail trade/Automotive and motorcycle repair/Accommodation and food services	1.33	1.12–1.57
Transportation and warehousing/Information and communication	0.85	0.68–1.06
Professional, financial and business services	0.96	0.77–1.19
Healthcare and social assistance	1.22	0.97–1.53
Education services/Public administration, social security	1.07	0.87–1.32
Other public and personal services	0.99	0.78–1.25
Occupational position		
Top and middle manager	0.65	0.46–0.94
White collar	0.94	0.69–1.28
Blue collar	1.02	0.74–1.40
Apprentice or other type of employment	ref	
Working hours		
1–34 h/week	1.22	1.07–1.41
35–40 h/week	ref	
41–48 h/week	1.12	0.92–1.36
> =49 h/week	0.87	0.68–1.13
Shift work		
No	ref	
Yes	1.21	1.07–1.36
Biomechanical and Ergonomic risk		
No Risk Perceived/No Risk Detected	ref	
Risk Perceived /Risk Detected	1.63	1.36–1.94
No Risk Perceived/Risk Detected	1.16	0.91–1.47
Risk Perceived /No Risk Detected	1.45	1.28–1.65
Video Display Terminal risk		
No Risk Perceived/No Risk Detected	ref	
Risk Perceived /Risk Detected	0.79	0.67–0.92
No Risk Perceived/Risk Detected	0.72	0.57–0.90
Risk Perceived /No Risk Detected	0.97	0.84–1.12
Work-related stress		
Low	ref	
Moderate	1.20	1.03–1.41
High	1.45	1.22–1.73
Very high	1.75	1.47–2.08
Anxiety		
No	ref	
Yes	1.70	1.48–1.96
Depression		
No	ref	
Yes	1.35	1.12–1.64
Insomnia		
No	ref	
Yes	1.62	1.44–1.84

**Table 4:** Multivariate logistic regression analysis assessing factors associated with the occurrence of Shoulders, neck and/or upper limb pain in a representative sample of 8000 Italian workers

Variable	OR	95% CI
Sex		
Male	ref	
Female	2.00	1.80–2.22
Age		
19–24	0.96	0.75–1.22
25–34	1.06	0.92–1.21
35–44	ref	
45–54	1.16	1.03–1.31
55–64	1.22	1.05–1.43
Educational level		
Lower secondary	ref	
Upper secondary	0.78	0.69–0.89
Graduate and post graduate	0.67	0.57–0.78
BMI		
Underweight	1.33	0.95–1.87
Normal	ref	
Overweight	1.17	1.05–1.30
Obese	1.34	1.12–1.61
Firm size		
1 to 9	ref	
10 to 49	1.11	0.95–1.31
50–249	1.22	1.04–1.44
≥ 250	1.16	1.00–1.35
Biomechanical and Ergonomic risk		
No Risk Perceived/No Risk Detected	ref	
Risk Perceived /Risk Detected	1.70	1.44–1.99
No Risk Perceived/Risk Detected	0.86	0.70–1.07
Risk Perceived /No Risk Detected	1.60	1.43–1.79
Video Display Terminal risk		
No Risk Perceived/No Risk Detected	ref	
Risk Perceived /Risk Detected	1.02	0.89–1.16
No Risk Perceived/Risk Detected	0.79	0.65–0.96
Risk Perceived /No Risk Detected	1.00	0.88–1.13
Work-related stress		
Low	ref	
Moderate	1.22	1.07–1.40
High	1.44	1.24–1.67
Very high	1.78	1.53–2.08
Anxiety		
No	ref	
Yes	1.54	1.34–1.77
Depression		
No	ref	
Yes	1.36	1.12–1.65
Insomnia		
No	ref	
Yes	1.75	1.56–1.97
Training on health and safety at work		
Yes	0.82	0.73–0.92
No	ref	

### Risk perception vs risk assessed at work and MSDs

As shown in Tables 2, 3 and 4, Risk Perceived vs Risk Detected for Biomechanical and Ergonomic risk and VDU risk provided interesting findings since showed strong association with all three outcomes of MSDs. In particular, participants perceiving exposure to Biomechanical and Ergonomic risk and which are included in a health surveillance programme (Risk Perceived and Risk Detected) were significantly more likely to experience either Back (OR: 1.91; 95%CI: 1.62–2.24), Lower limbs (OR: 1.63; 95%CI: 1.36–1.94) or Shoulders, neck and/or upper limbs (OR: 1.70; 95%CI: 1.44–1.99) compared to the reference category (No Risk Perceived and No Risk Detected). Surprisingly, also participants perceiving exposure to Biomechanical and Ergonomic risk but not included in a health surveillance programme for this risk (Risk Perceived and No Risk Detected) have had significantly higher odds of reporting MSDs, respectively Back pain (OR: 1.63; 95%CI: 1.46–1.82), Lower limb pain (OR:1.45; 95%CI: 1.28–1.65) and Shoulders, neck and/or upper limb pain (OR: 1.60; 95%CI: 1.43–1.79).

Findings revealed a strong association also for VDU Risk Perceived vs VDU Risk Detected, and MSDs to Back and Lower limbs. In particular, participants perceiving no exposure to VDU risk but who are conversely included in a health surveillance programme for this risk (No Risk Perceived and Risk Detected) were less likely to experience either Back (OR:0.67; 95%CI: 0.55–0.81) or Lower limb pain (OR:0.72; 95%CI: 0.57–0.90). A lower probability of reporting Lower limb pain was also observed among participants perceiving exposure to VDU risk and which are included in a health surveillance programme for such risk (Risk Perceived and Risk Detected; OR:0.79; 95%CI: 0.67–0.92). No evidence of association was found for VDU Risk Perceived vs VDU Risk Detected and Shoulders, neck and/or upper limbs pain.

An in depth description of such aforementioned 4 groups emerged by crossing Risk Perceived and Risk Detected for each risk, with particular reference to the group Risk Perceived /No Risk Detected, is reported in Tables 5, 6, 7, 8, 9 and 10. We found a significantly higher proportion of participants perceiving exposure to Biomechanical and Ergonomic risk but not included in a health surveillance programme for such risk (Risk Perceived and No Risk Detected) in the age ranging from 19 to 24 (39.9%). Main sector is Transportation and warehousing/Information and communication (38.9%) and they come mainly from companies with more than 250 employees (35.8%). With regard to VDU risk, a significantly higher proportion of respondents perceiving to be exposed to this risk but not included in a health surveillance programme (Risk Perceived/No Risk Detected) was found in the age class ranging from 45 to 54 (24.5%), in the Professional, financial and business services (38.0%) and in those working in companies with more than 250 employees (25.6%).

**Tables 5:** Risk Perceived and Risk Detected for Biomechanical and Ergonomic: Comparison for Age

Biomechanical and Ergonomic risk	Age									
	19–24		25–34		35–44		45–54		55–64	
	N (%)	Res.	N (%)	Res.	N (%)	Res.	N (%)	Res.	N (%)	Res.
No Risk Perceived/No Risk Detected	159 (42.3)	−2.7	747 (48.4)	−0.6	1209 (47.1)	−2.4	1202 (49.8)	0.8	610 (55.5)	4.5
Risk Perceived /Risk Detected	37 (9.8)	−1.1	174 (11.3)	−0.6	344 (13.4)	3.3	280 (11.6)	−0.2	100 (9.1)	−2.9
No Risk Perceived/Risk Detected	30 (8.0)	1.4	99 (6.4)	0.3	148 (5.8)	−1.2	160 (6.6)	1.0	61 (5.6)	−1.0
Risk Perceived /No Risk Detected	150 (39.9)	2.9	523 (33.9)	0.9	865 (33.7)	1.0	771 (32.0)	−1.3	329 (29.9)	−2.3

**Table 6:** Risk Perceived and Risk Detected for Biomechanical and Ergonomic: Comparison for Sector

Biomechanical and Ergonomic risk	Agriculture, fishing, and hunting		Manufact./ Primary industry/ Mining/ Utilities		Construction		Wholesale and retail trade/ Automotive and motorcycle repair/ Accommodation and food services		Transport. and warehousing/ Information and communication		Professional/ financial and business services	Healthcare and social assistance		Education services/ Public administration, social security		Other public and personal services		
	N (%)	Res.	N (%)	Res.	N (%)	Res.	N (%)	Res.	N (%)	Res.	N (%)	Res.	N (%)	Res.	N (%)	Res.	N (%)	Res.
No Risk Perceived/No Risk Detected	77 (45.0)	−1.1	796 (42.6)	−6.4	178 (840.9)	−3.5	753 (53.5)	3.7	297 (45.8)	−1.8	571 (60.1)	7.2	238 (33.6)	−8.6	705 (58.5)	7.1	312 (51.6)	1.3
Risk Perceived /Risk Detected	22 (12.9)	0.5	351 (18.8)	10.9	77 (17.7)	4.0	141 (10.0)	−2.1	70 (10.8)	−0.7	25 (2.6)	−9.3	154 (21.8)	8.7	36 (3.0)	−10.2	59 (9.8)	−1.5
No Risk Perceived/Risk Detected	17 (9.9)	2.0	177 (89.5)	6.6	44 (10.1)	3.5	91 (6.5)	0.4	29 (4.5)	−1.9	25 (2.6)	−4.9	50 (7.1)	1.0	31 (2.6)	−5.7	34 (5.6)	−0.6
Risk Perceived /No Risk Detected	55 (32.2)	−0.2	543 (29.1)	−4.1	136 (31.3)	−0.8	422 (30.0)	−2.6	253 (39.0)	3.4	329 (34.6)	1.2	266 (37.6)	2.7	434 (36.0)	2.4	200 (33.1)	0.0

**Table 7:** Risk Perceived and Risk Detected for Biomechanical and Ergonomic: Comparison for Firm size

Biomechanical and Ergonomic risk	Firm size							
	1 to 9		10 to 49	50 to 249			≥ 250	
	N (%)	Res.	N (%)	Res.	N (%)	Res.	N (%)	Res.
No Risk Perceived/No Risk Detected	715 (57.2)	6.4	810 (51.7)	2.5	832 (48.6)	−0.3	1388 (44.3)	−6.7
Risk Perceived /Risk Detected	88 (7.0)	−5.8	189 (12.1)	0.2	201 (11.7)	−0.3	435 (13.9)	4.4
No Risk Perceived/Risk Detected	65 (5.2)	−1.6	118 (7.5)	2.5	103 (6.0)	−0.36	189 (6.0)	−0.5
Risk Perceived /No Risk Detected	383 (30.6)	−2	449 (28.7)	−4.1	577 (33.7)	0.6	1124 (35.8)	4.3

**Tables 8:** Risk Perceived and Risk Detected for Video Display Terminal risk: Comparison for Age

Video Display Terminal risk	Age									
	19–24		25–34		35–44		45–54		55–64	
	N (%)	Res.	N (%)	Res.	N (%)	Res.	N (%)	Res.	N (%)	Res.
No Risk Perceived/No Risk Detected	208 (55.3)	4.2	699 (45.3)	0.4	1173 (45.7)	1.1	1029 (42.6)	−2.6	477 (43.4)	−1.1
Risk Perceived /Risk Detected	61 (16.2)	−3.4	354 (22.9)	−0.5	608 (23.7)	0.3	603 (25.0)	2.1	251 (22.8)	−0.5
No Risk Perceived/Risk Detected	17 (4.5)	−2.5	111 (7.2)	−1.2	212 (8.3)	0.7	191 (7.9)	−0.1	105 (9.6)	2.1
Risk Perceived /No Risk Detected	90 (23.9)	0.1	379 (24.6)	0.8	573 (22.3)	−2.0	590 (24.5)	1.0	267 (24.3)	0.4

**Tables 9:** Risk Perceived and Risk Detected for Video Display Terminal risk: Comparison for Sector

Video Display Terminal risk	Agriculture. fishing. and hunting		Manufacturing/ Primary industry/ Mining/ Utilities		Construction		Wholesale and retail trade/ Automotive and motorcycle repair/ Accommodation and food services		Transportation and warehousing/ Information and communication		Professional. financial and business services		Healthcare and social assistance		Education services/ Public administration. Social security		Other public and personal services	
	N (%)	Res.	N (%)	Res.	N (%)	Res.	N (%)	Res.	N (%)	Res.	N (%)	Res.	N (%)	Res.	N (%)	Res.	N (%)	Res.
No Risk Perceived/No Risk Detected	102 (59.7)	3.9	952 (51.0)	6.1	255 (58.6)	5.9	798 (56.7)	9.9	267 (41.1)	−2.0	165 (17.4)	−18.1	301 (42.5)	−1.3	473 (39.2)	−4.3	273 (45.1)	0.1
Risk Perceived /Risk Detected	39 (22.8)	−0.2	441 (23.6)	0.2	80 (18.4)	−2.6	196 (13.9)	−9.3	195 (30.1)	4.1	349 (36.7)	10.3	156 (22.0)	−0.9	278 (23.1)	−0.4	143 (23.6)	0.1
No Risk Perceived/Risk Detected	10 (5.9)	−1.0	188 (10.1)	3.9	33 (7.6)	−0.3	107 (7.6)	−0.5	48 (7.4)	−0.5	77 (8.1)	0.2	55 (7.8)	−0.2	71 (5.9)	−2.9	47 (7.8)	−0.2
Risk Perceived /No Risk Detected	20 (11.7)	−3.7	286 (15.3)	−9.8	67 (15.4)	−4.2	306 (21.8)	−1.9	139 (21.4)	−1.5	359 (38.0)	10.8	196 (27.7)	2.6	384 (31.8)	7.2	142 (23.5)	−0.2

**Tables 10:** Risk Perceived and Risk Detected for Video Display Terminal risk: Comparison for Firm size

Video Display Terminal risk	Firm size							
	1 to 9		10 to 49		50 to 249		≥ 250	
	N (%)	Res.	N (%)	Res.	N (%)	Res.	N (%)	Res.
No Risk Perceived/No Risk Detected	732 (58.5)	11.2	798 (51.0)	6.1	771 (45.0)	0.8	1081 (34.5)	−14.2
Risk Perceived /Risk Detected	139 (11.1)	−11.6	322 (20.6)	−3.5	389 (22.7)	−1.4	987 (31.5)	12.8
No Risk Perceived/Risk Detected	80 (6.4)	−2.4	139 (8.9)	1.3	135 (7.9)	−0.4	266 (8.5)	1.1
Risk Perceived /No Risk Detected	300 (24.0)	0.1	307 (19.6)	−4.4	418 (24.4)	0.6	802 (25.6)	3.0

### Sociodemographic characteristics and MSDs

All control variables (age, sex, educational level and BMI) were found to be strongly associated with the presence of MSDs (Tables 2, 3 and 4). At a glance, the probability of experiencing either low Back, Lower limb or Shoulders, neck and/or upper limb pains increases with age and is maximum in the age ranging from 55 to 64 years (respectively OR: 1.32; 95%CI: 1.13–1.55; OR: 1.95; 95%CI: 1.64–2.32; OR: 1.22; 95%CI: 1.05–1.43). Females are more likely to report MSDs than males and this is particularly evident for Shoulders, neck and/or upper limb pain (OR: 1.45, 95%CI: 1.31–1.61; OR: 1.23, 95%CI: 1.08–1.41; OR: 2.00, 95%CI: 1.80–2.22). Higher educational attainment is associated with a 20 to 30% lower probability of all MSDs. Finally, high BMI (overweight and obesity) is associated with an increased likelihood of experiencing MSDs, as especially evident for Lower limbs (OR: 1.38, 95%CI: 1.22–1.55; OR: 2.37, 95%CI: 1.96–2.86).

### Occupational characteristics and MSDs

As regard to the occupational aspects investigated by this study, significant associations were found among Lower limb pain and the following factors: occupational sector, occupational position, working hours and shift work (Table 3). In particular, working in Commerce (OR: 1.33; 95%CI: 1.12–1.57), working less than 34 h per week (OR: 1.22; 95%CI: 1.07–1.41) and shift work (OR: 1.21; 95%CI: 1.07–1.36) were found to be strongly associated with a higher probability of reporting Lower limb pain; a lower probability of Lower limb pain was found for top and middle managers (OR: 0.65; 95%CI: 0.46–0.94).

### Other health related aspects and MSDs

With regard to health related conditions (Tables 2, 3 and 4), results of multivariable logistic regression analyses revealed that those reporting from moderate to high level of work-related stress (OR and 95%CI for each category: 1.44, 1.26–1.64; 1.50, 1.30–1.74; 2.03; 1.75–2.36), anxiety (OR: 1.50; 95%CI: 1.31–1.73) and insomnia (OR: 1.78; 95%CI: 1.59–2.0) had significantly higher odds of experiencing Back pain. Similar associations were found between these factors and both Lower limb and Shoulders, neck and/or upper limb pains. In addition, depression was found to be significantly associated with both Lower limb (OR: 1.35; 95%CI: 1.12–1.64) and Shoulders, neck and/or upper limb pain (OR: 1.36; 95%CI: 1.12–1.65). Concerning the management of health and safety at work, having received training in health and safety was associated with lower odds of experiencing either Back (OR: 0.84; 95%CI: 0.75–0.94) or Shoulders, neck and/or upper limbs pain (OR: 0.82; 95% CI: 0.73–0.92), while no evidence of association emerged for lower limb pain.

## Discussion

Although the prevalence of WMSDs and their related management have been extensively investigated, yet important gaps in surveillance systems remain, and a significant proportion of the global working population continues to live and work with these disabling conditions. In the present study, we have focused on a representative sample of the Italian working population, assessing their exposure perception to the biomechanical/ergonomic and VDU risks and the actual health surveillance programs for these two risks proving strong association with WMSDs.

The assessed prevalence of low Back pain, Shoulders, neck and/or upper limbs pain and Lower limb pains assessed in this study are in line with the literature. Indeed, WMSDs are the second most common cause of disability worldwide measured by YLDs, with low back pain ranking as the most frequent condition [21].

Some sociodemographic variables (age, sex, educational level and BMI) seem to be closely associated with the presence of WMSDs confirming available evidence. The WMSD prevalence increases with age and reach its maximum in the range between 55 and 64 years being consistent with data by the EU- OSHA. Indeed, in this age range the number of self-reported symptoms is 1.7 times higher than the range of 25–34 [8]. The recorded evidence of increasing WMSDs with advanced age may be related to the fact that older workers are becoming more prevalent in the work force due to improvement in health, increased life expectancy and a higher rate of employment in companies [22]. Women reporting WMSDs more frequently than men also agrees with evidence observed in other previous studies [23]. Indeed, women who do the same job tasks as men often face a higher risk of WMSDs that may be due to both biological divergences as well as differences in social roles, activities and behaviors [23]. Moreover, they have a moderately increased risk of chronicity compared to men [24]. Education is likely a protective factor for WMSDs that may be due to the association between higher level of education and working position and therefore a decreased risk for these disorders. Farioli et al. showed that the prevalence of back and upper limb pain increased with age and was higher among women and workers with lower educational level in a population of 43,816 subjects from 34 European countries [25]. Regarding BMI, it is widely acknowledged that obese or overweight workers have a greater risk of developing WMSDs, as they are more susceptible to risks from vibrations, repetitive movements and manual handling of loads [26]. Obese workers are twice as likely as normal weight workers to develop upper limb tendinopathies and four times as likely to develop carpal tunnel syndrome. In accordance to our data, BMI was also associated with musculoskeletal symptoms of the lower extremity [27]. Furthermore, obesity markedly increases the risk of disability retirement due to WMSDs [28].

The strong association between work in the commerce sector and leg pain could possibly be linked to the long time work standing in this sector (shop assistants, waiters). The prolonged standing work posture has a strong association with pain in feet and legs, with the onset of varicose veins, and chronic venous insufficiency [29].

Among the others, most interesting findings emerged by our study are those related to the risk perception compared to risk detection linked to MSDs. Such findings showed that workers perceiving exposure to biomechanical/ergonomic and VDU risks, but not included in a health surveillance program for these occupational risk factors (Risk Perceived/No Risk Detected), had significantly higher odds of reporting MSDs, similarly to those perceiving exposure and included in a health surveillance program (Risk Perceived/ Risk Detected). These results were unexpected and would suggest a rather high risk profile (referring to biomechanical/ergonomic and/or VDU risks) for these subjects, at least similar to that of workers undergoing health surveillance medical examinations. However, it is important to underline that the items of questionnaire measuring the occurrence of WMSDs did not provide for the detection of a time frame and consequently it should be considered that workers not included in a health surveillance program may have had WMSDs for only a short period of time, whereas workers included in the program possibly had chronic complaints. Therefore, this result should be considered with caution and further investigated in future using appropriately designed and targeted studies. Nevertheless, it is still possible to hypothesize that the OSH management system may not have been able to intercept them, thus highlighting some potential critical issues in the evaluation and management process of these specific occupational risk factors. There are several possible reasons that can explain this finding and probably each of them contributes partially, but synergistically, in determining the evidence here reported. Obviously, considering the cross-sectional design of our study, we are not able to determine or identify causal correlations between the results obtained and any underlying determinants but, on the basis of the observed associations, we can still try to put forward plausible hypotheses. First of all, it should be considered that the available evaluation tools and strategies applied for biomechanical/ergonomic risk assessment and management are based on observational methods that require specific competencies and adequate training in order to be selected and used properly. For example in this regard, among the numerous tools available, there is no single one that is suitable for all purposes and consequently the choice of the most appropriate method may be quite challenging [14]. Moreover, the final users (i.e. occupational physicians or OSH technicians) of these evaluation instruments could have a limited knowledge since the operating indications are often provided in unfamiliar language or the methods have been developed for a specific work sector and their translation into a different professional context is complex and not always reliable [14]. Therefore, an initial possible explanation of this result could lie in a potential incorrect application or inadequate biomechanical/ergonomic risk assessment by the OSH management system.

Moreover, it is important to keep in mind that WMSDs are associated with numerous and different risk factors, some of which are socio-demographic (e.g. age, sex, BMI, leisure physical activity) but others are closely related to the working activities carried out [30]. In fact, it is no coincidence that MSDs prevalence is significantly higher in workers compared to general population since several workplace hazard exposures such as manual handling of heavy loads, awkward postures, hand-arm and whole body mechanical vibrations, repetitive movements may play an important role in their onset [31, 32]. Most importantly, in many industrial sectors the damaging action of these occupational risk factors occurs at the same time on the same target organ as the working activities involve a simultaneous exposure of the worker. Therefore, the case could arise in which the single assessment of each of the aforementioned risk factors highlights a controlled risk condition with exposure levels that are below the values that require the application of secondary prevention measures such as the health surveillance. Consequently, these workers are probably not subjected to health surveillance medical examinations (as the risk assessment and management system would not provide for its activation) but, in the long run, chronic and simultaneous exposure to multiple and synergic occupational risk factors might determine the occurrence of WMSDs. These complex situations involving multiple exposures to risk factors with potential synergistic action should be carefully evaluated by the OSH management system also in consideration of the results observed in our study.

Indeed, regarding the biomechanical/ergonomic risk, workers who perceive the risk but are not included in a health surveillance program for such risk (Risk Perceived and No Risk Detected) are mainly employed in transportation, warehousing/information and communication that are particular working activities often characterized by the simultaneous exposure to awkward postures, mechanical vibration, manual handling of heavy loads and repetitive movements. Furthermore, they are mainly in the age range of 19–24 years and this may be due to the precarious employment conditions, such as short-term contracts or low-wage work, which are more common among younger workers in Europe [15] and seem to be related to lower standards of OSH protection [16]. In this regard, it should be considered that younger workers have less access and a lower awareness of OHS issues compared with older workers, therefore, constituting an important challenge for OSH systems [4].

With regard to VDU risk, these workers (Risk Perceived/No Risk Detected) are in the age range of 45–54 years and they are mainly involved in the professional, financial and business services. Concerning the higher OR of workers who, although not included in the health surveillance system for exposure to VDU risk factor, perceived this exposure as high, it is noteworthy that according to regulatory framework currently in force in Italy VDU worker spend at least 20 h of their weekly working time using a VDU unit. Usually, the assessment of this working time is carried out by means of validate questionnaire and/or checklists [13]. However, even if a subject is not classified as a VDU worker it could happen that its actual exposure time to the VDU unit is significantly higher (often >20 h). Furthermore, also in this case, this type of work involves exposure to additional risk factors (i.e. awkward postures and repetitive movements of the upper limbs) closely related to WMSDs. This hypothesis would be further corroborated by the observed positive and statistically significant association between Lower limb pain and working hours. Moreover, it is possible to speculate that, if these subjects are not actually classified as VDU workers, then also the attention paid to them by the OSH management system, both in terms of prevention and protection measures and training and information programs, can be significantly lower than their VDU colleagues.

WMSDs often pose also major threats to mental health and can be associated with increased risk of developing other chronic health conditions [33]. Data from our study also support the correlation between psychosocial factors and MSDs, already highlighted in other studies. Indeed, Sobeih et al. [34] conducted a systematic review examining the link between psychosocial factors and the presence of WMSDs among construction workers. All studies reported a correlation between WMSDs and at least one psychosocial factor, the most frequent being work-related stress, poor professional satisfaction, low control over work and pressing demands in terms of work performance. Leka et al. [35] identified 16 studies describing the existence of a link between psychosocial factors (including stress, long working hours, no control over their work, lack of social support) and onset of WMSDs including injury from biomechanical overload (muscle injury due to frequent use of the same muscles) and pain in the upper limbs, neck and back. A correlation between WMSDs and anxiety and depression was observed by Magnavita et al. [36] on caregiving workers. The authors showed, that Back pain was associated with workload, depression, age and anxiety; cervical pain was associated with psychosocial factors of stress, female sex and anxiety.

Moreover, in the present study, training in health and safety was found to be a protective factor for Back pain and Upper limb pain confirming the crucial role of education and training. However, it is currently agreed that training alone is not enough, prevention plans need an overall strategy, that must take into account all the aspects that influence the biomechanical risk: organizational (quantity of operators; definition of procedures; working times; working relationships), technical-structural (availability and quality of tools/aids; typology and organization of work spaces) and cultural (adequate training and knowledge of handling procedures and techniques). Over time, only global strategic interventions have proven to be able to adequately manage the risk for operators, reducing disease, absences and reflected costs. Other partial interventions, such as the training of healthcare operators have shown enormous limits of effectiveness, making the relative economic investments fruitless [37].

All in all, the main strength of this study is that this allows a comparison among workers’perceptions of exposure to some occupational risks, their actual involvement in health surveillance program, and the emergence of MSDs. Few previous studies focused on the effectiveness of OSH management system in detecting risks associated to MSDs. Thank to our results we offered thus insights and reflections on tools and methods used for the evaluation to contribute in improving the OSH risk management in organizations.

Some limitations of this study must be addressed too with a view to future improvements. First, the cross-sectional design allows us to describe associations but not drawing causal inferences about the effects of the different variables on MSDs. Nevertheless, data collected are part of a national survey (INSuLa) and are based on a large representative sample, that represents a strength of this study since allows to obtain valid results, which adequately reflect population distribution. Moreover, this survey is now becoming a monitoring system to follow changes over time. A new wave was already conducted in 2019 on further 8000 workers and preliminary data will be presented in 2020. As second limitation is related to the self-reporting of measures. Particularly, data on MSDs are self-report and refers to the last 12 months, thus might tend to have a recall biased, namely error that can occur when participants do not remember their experiences accurately or omit detail. Combining self-report data with other information by using a multi-method assessment provides more likely accurate data on the outcome. Thus, ongoing studies are focussing on collecting self report data and integrate such information with medical examination outcomes or diagnosed information.

## Conclusion

Our findings confirm that WMSDs are extremely important and currently represent a stimulating and complex health challenge for occupational medicine since a significant number of workers experience one or more WMSDs in carrying out their job activities. Interestingly, we have noted that the WSMDs prevalence observed in workers undergoing health surveillance medical examinations for the main risk factors involved in the onset of these diseases was very similar to that found in workers who, although reported a high risk perception for the aforementioned occupational risk factors, were not subjected to the health surveillance system.

In this regard, we have tried to provide some plausible explanations of this possible WMSDs underestimation but in any case these assumptions should be considered with caution and carefully evaluated since the verification of the adequacy of the biomechanical/ergonomic risk assessment process was not the main purpose of the survey from which the results here reported were obtained. Nevertheless, in our opinion these findings provide some interesting insights that obviously should be further investigated by future studies focusing on the analysis of the effectiveness of current evaluation methodologies and strategies that are commonly used to assess biomechanical/ergonomic risk factors. In this regard, novel approaches to tackle this topic are desirable in order to drive systems and services toward high-value care. These innovative strategies should be mainly aimed at improving our understanding of emerging occupational risks that may contribute to WMSDs, identifying gaps in current evaluation methodologies and investigating the effectiveness and quality of preventive workplace interventions. Hence, the ultimate goal is to improve health surveillance systems by obtaining more compelling and accurate data for policy makers.

## Data Availability

The data that support the findings of this study are available from INAIL but restrictions are applied to the availability of these data, which were used under license for the current study, and so are not publicly available. Data are however available from the authors upon reasonable request and with permission of INAIL.

## References

[1] Briggs AM, Woolf AD, Dreinhofer K, Homb N, Hoy DG, Kopansky-Giles D (2018). Reducing the global burden of musculoskeletal conditions. Bull World Health Organ.

[2] Disease GBD, Injury I, Prevalence C (2018). Global, regional, and national incidence, prevalence, and years lived with disability for 354 diseases and injuries for 195 countries and territories, 1990-2017: a systematic analysis for the global burden of Disease study 2017. Lancet..

[3] Hagberg M, Violante FS, Bonfiglioli R, Descatha A, Gold J, Evanoff B (2012). Prevention of musculoskeletal disorders in workers: classification and health surveillance - statements of the Scientific Committee on Musculoskeletal Disorders of the International Commission on Occupational Health. BMC Musculoskelet Disord.

[4] Sim J, Lacey RJ, Lewis M (2006). The impact of workplace risk factors on the occurrence of neck and upper limb pain: a general population study. BMC Public Health.

[5] Hossain MD, Aftab A, Al Imam MH, Mahmud I, Chowdhury IA, Kabir RI (2018). Prevalence of work related musculoskeletal disorders (WMSDs) and ergonomic risk assessment among readymade garment workers of Bangladesh: a cross sectional study. PLoS One.

[6] Ahn G, Hur S, Jung MC (2020). Bayesian network model to diagnose WMSDs with working characteristics. Int J Occup Saf Ergon.

[7] Communication from the Commission to the European Parliament tC, the European Economic and Social Committee and the Committee of the Regions, ‘An EU Strategic Framework on Health and Safety at Work 2014–2020’, COM (2014) 332 final. Available at: https://eur-lex.europa.eu/legal-content/EN/TXT/?uri=COM:2014:332:FIN (Accessed on 08 May, 2020). [.

[8] European Agency for Safety and Health at Work (2019). Work-related musculoskeletal disorders: prevalence, costs and demographics in the EU. European Risk Observatory Report.

[9] European Foundation for the Improvement of Living and Working Conditions (2015). Sixth European Working Conditions Survey.

[10] Relazione annuale INAIL 2018. Scheda / 1 - Infortuni e malattie professionali: i dati del 2018. Available at https://www.inail.it/cs/internet/docs/all-relazione-annuale-inail-2018-scheda-1-infortuni-mp.pdf (Accessed on 08 May, 2020).

[11] Italian Workers’ Compensation Authority (2014). National surveys on health and safety at work (INSuLa Project): Employees and Employers.

[12] European Agency for Safety and Health at Work. Work-related musculoskeletal disorders: prevalence, costs and demographics in the EU, National report: Italy. Available at: https://osha.europa.eu/sites/default/files/publications/documents/work_related_MSDs_Italy.pdf (Accessed on 24 May, 2020).

[13] Ricco M, Cattani S, Gualerzi G, Signorelli C (2016). Work with visual display units and musculoskeletal disorders: a cross-sectional study. Med Pr.

[14] Takala EP, Pehkonen I, Forsman M, Hansson GA, Mathiassen SE, Neumann WP (2010). Systematic evaluation of observational methods assessing biomechanical exposures at work. Scand J Work Environ Health.

[15] European Agency for Safety and Health at Work. Directive 90/269/EEC - manual handling of loads. Available at: https://eur-lex.europa.eu/legal-content/EN/TXT/?uri=CELEX:01990L0269-20070627 (Accessed on 24 May, 2020).

[16] Armstrong TJ, Burdorf A, Descatha A, Farioli A, Graf M, Horie S (2018). Scientific basis of ISO standards on biomechanical risk factors. Scand J Work Environ Health.

[17] European Agency for Safety and Health at Work. Directive 90/270/EEC - display screen equipment. Available at: https://eur-lex.europa.eu/legal-content/EN/TXT/?uri=CELEX:01990L0270-20070627 (Accessed on 24 May, 2020).

[18] Mikkelsen S, Vilstrup I, Lassen CF, Kryger AI, Thomsen JF, Andersen JH (2007). Validity of questionnaire self-reports on computer, mouse and keyboard usage during a four-week period. Occup Environ Med.

[19] Zhang Z (2016). Variable selection with stepwise and best subset approaches. Ann Transl Med.

[20] Chowdhury MZI, Turin TC (2020). Variable selection strategies and its importance in clinical prediction modelling. Fam Med Community Health.

[21] Hoy D, March L, Brooks P, Blyth F, Woolf A, Bain C (2014). The global burden of low back pain: estimates from the global burden of Disease 2010 study. Ann Rheum Dis.

[22] Olanre Okunribido TW. Ageing and work-related musculoskeletal disorders: a review of the recent literature. Health Safety Executive. 2010.

[23] Guignon N (2008). ‘Risques professionnels : les femmes sont-elles à l’abri ?’, in Femmes et hommes – Regards sur la parité.

[24] Gjesdal S, Bratberg E, Maeland JG (2011). Gender differences in disability after sickness absence with musculoskeletal disorders: five-year prospective study of 37,942 women and 26,307 men. BMC Musculoskelet Disord.

[25] Farioli A, Mattioli S, Quaglieri A, Curti S, Violante FS, Coggon D (2014). Musculoskeletal pain in Europe: the role of personal, occupational, and social risk factors. Scand J Work Environ Health.

[26] Di Tecco C, Fontana L, Adamo G, Petyx M, Iavicoli S (2020). Gender differences and occupational factors for the risk of obesity in the Italian working population. BMC Public Health.

[27] Viester L, Verhagen EA, Oude Hengel KM, Koppes LL, van der Beek AJ, Bongers PM (2013). The relation between body mass index and musculoskeletal symptoms in the working population. BMC Musculoskelet Disord.

[28] Shiri R, Falah-Hassani K, Lallukka T (2020). Body mass index and the risk of disability retirement: a systematic review and meta-analysis. Occup Environ Med.

[29] Messing KL, Katherine, Laperrière È, Thibault M (2018). Pain Associated with Prolonged Constrained Standing: The Invisible Epidemic.

[30] Dong H, Zhang Q, Liu G, Shao T, Xu Y (2019). Prevalence and associated factors of musculoskeletal disorders among Chinese healthcare professionals working in tertiary hospitals: a cross-sectional study. BMC Musculoskelet Disord.

[31] Grieco A, Molteni G, De Vito G, Sias N (1998). Epidemiology of musculoskeletal disorders due to biomechanical overload. Ergonomics..

[32] Malchaire J, Cock N, Vergracht S (2001). Review of the factors associated with musculoskeletal problems in epidemiological studies. Int Arch Occup Environ Health.

[33] Briggs AM, Cross MJ, Hoy DG, Sanchez-Riera L, Blyth FM, Woolf AD (2016). Musculoskeletal health conditions represent a global threat to healthy aging: a report for the 2015 World Health Organization world report on ageing and health. Gerontologist..

[34] Sobeih T, Salem S, Genaidy A, Daraiseh N, Shell R (2006). Psychosocial factors and musculoskeletal disorders in the construction industry: a systematic review. Theor Issues Ergon Sci.

[35] Leka S, Jain A (2010). Health impact of psychosocial hazards at work: an overview - World Health Organization.

[36] Magnavita N, Elovainio M, De Nardis I, Heponiemi T, Bergamaschi A (2011). Environmental discomfort and musculoskeletal disorders. Occup Med (Lond).

[37] Robson LS, Stephenson CM, Schulte PA, Amick BC, Irvin EL, Eggerth DE (2012). A systematic review of the effectiveness of occupational health and safety training. Scand J Work Environ Health.

